# Leaf Structural, Physiological and Biochemical Responses to Contrasting Light Environments in *Iris pumila* L.: Evidence from a Reciprocal Transplant Experiment

**DOI:** 10.3390/plants15071009

**Published:** 2026-03-25

**Authors:** Sanja Manitašević Jovanović, Ana Vuleta

**Affiliations:** Department of Evolutionary Biology, Institute for Biological Research “Siniša Stanković”-National Institute of the Republic of Serbia, University of Belgrade, 11000 Belgrade, Serbia; ana.vuleta@ibiss.bg.ac.rs

**Keywords:** *Iris pumila* L., light intensity, phenotypic plasticity, population differentiation, reciprocal transplant experiment

## Abstract

Light availability is a key environmental factor influencing plant functional traits and ecological strategies. To investigate how natural populations of *Iris pumila* respond to contrasting irradiance, we conducted an *in situ* reciprocal transplant experiment using clonal genotypes from two natural populations, each originating from an open dune and a shaded forest habitat. Leaves collected from each of the replanted and transplanted genotypes were analyzed for structural (specific leaf area—SLA, leaf dry matter content—LDMC), physiological (specific leaf water content—SLWC, photosynthetic pigments) and biochemical (peroxidase—POD, glutathione reductase—GR, phenolics and anthocyanins) traits. Shade-grown individuals developed thinner leaves with higher SLA and chlorophyll content, enhancing light-harvesting efficiency, whereas sun-exposed plants exhibited greater LDMC, increased POD and GR activities and higher anthocyanin levels—traits consistent with enhanced photoprotection under high irradiance. All genotypes exhibited pronounced plasticity to light intensity, with habitat exerting a stronger influence on trait expression than population origin. To evaluate oxidative balance, we proposed the ODAC index (Oxidative Damage to Antioxidant Capacity), which integrates lipid peroxidation with antioxidant capacity. ODAC values revealed consistent population-level differences, with higher values in Dune genotypes across habitats, indicating a constitutively elevated oxidative load relative to antioxidant protection and suggesting differentiation in redox regulation between populations. Overall, leaf trait variation in *I. pumila* appears to be primarily driven by plastic responses to light conditions, while differentiation in oxidative physiology contributes to functional divergence between populations.

## 1. Introduction

Natural habitats are shaped by a wide array of physical, chemical and biological factors, whose spatial and temporal variability drives both short-term ecological and long-term evolutionary responses. For sessile organisms such as plants, successful adjustment to the heterogeneous distribution of essential environmental resources, including light, nutrients and water, represents a major ecological challenge [1,2]. Because plants are constantly exposed to environmental variability in space and time, their ability to adjust structural and physiological traits in response to fluctuating conditions is considered a key adaptive strategy. This flexibility, known as phenotypic plasticity, enables a single genotype to produce alternative phenotypes better suited to the prevailing environment [3,4]. Current perspectives emphasize that trait-based studies of phenotypic plasticity gain explanatory power when interpreted alongside emerging molecular insights into environmentally induced regulatory switches and their evolutionary consequences [5]. Environmental heterogeneity may occur along fine- or coarse-grained gradients, and this variation often determines whether plants evolve plastic (flexible) or constitutive (fixed) modes of adaptation [6,7,8].

Among ecological factors influencing plant performance, light represents one of the most variable environmental resources, providing a suitable context for exploring the adaptive significance of plasticity. Plants can modify their structural, physiological and biochemical traits to optimize light capture under contrasting irradiance levels [9,10,11,12,13]. Recent molecular and transcriptomic studies further show that shifts in irradiance and light quality (e.g., R:FR) trigger rapid, coordinated changes in gene expression underlying shade responses, providing a mechanistic link between environmental cues and leaf-level trait adjustment [14].

These adjustments are often reflected in leaf-level functional traits that play a central role in photosynthetic efficiency and overall plant fitness [15,16,17]. Two of the most informative and widely studied indicators of leaf structural adjustment are specific leaf area (SLA) and leaf dry matter content (LDMC). Numerous studies have demonstrated their strong predictive value for plant growth strategy, resource-use efficiency and environmental filtering across ecosystems [15,18]. SLA, defined as the ratio of leaf area to dry mass, reflects the balance between investment in light-harvesting surface and structural support, whereas LDMC, expressed as the proportion of dry mass relative to fresh mass, indicates tissue density and mechanical robustness [13,19]. High SLA values are typically associated with leaves developed under shaded or resource-rich conditions, where maximizing photosynthetic surface is advantageous, whereas lower SLA and higher LDMC characterize leaves formed under high irradiance, drought, or nutrient limitation, favoring thicker, denser and more stress-tolerant tissues [15,20,21].

Beyond structural adjustments, plants exhibit diverse physiological modifications that contribute to functional stability under fluctuating environmental conditions. Physiological traits such as specific leaf water content (SLWC) and the concentration and composition of photosynthetic pigments provide complementary insights into plant acclimation potential. SLWC indicates tissue hydration and turgor stability, which are essential for maintaining photosynthetic performance under stress [22,23]. Photosynthetic pigments, including chlorophylls (Chl) and carotenoids (Cars), reflect both the light-harvesting capacity and photoprotective status of the photosynthetic apparatus [24,25]. Shade-acclimated leaves typically exhibit a lower Chl *a*/*b* ratio and a higher total Chl content, whereas sun-acclimated leaves maintain a higher Chl *a*/*b* ratio and elevated carotenoid levels to enhance energy dissipation and protection against photooxidative stress [26,27,28].

In addition to structural and physiological adjustments, plants rely on a complex biochemical antioxidant network to mitigate oxidative stress caused by high irradiance, temperature extremes or drought [29]. Excess energy absorbed by the photosynthetic apparatus that cannot be efficiently used for photochemistry or dissipated as heat may lead to the overproduction of reactive oxygen species (ROS), which can damage cell membranes, proteins and nucleic acids [30,31,32,33]. Under moderate, non-stressful conditions, ROS act as important signaling molecules mediating growth, development and acclimation, but when their production exceeds antioxidant capacity, they become harmful. To maintain redox homeostasis, plants rely on a complex antioxidant network comprising both enzymatic and non-enzymatic components [34,35,36]. Within this network, peroxidase (POD) and glutathione reductase (GR) perform distinct yet complementary functions, acting in parallel pathways that collectively strengthen the plant’s antioxidant defense. POD contributes by catalyzing the oxidation of diverse phenolic substrates and detoxifying ROS [37], while GR maintains the pool of reduced glutathione—one of the most abundant cellular thiols that plays a central role in controlling ROS levels [38,39,40]. Non-enzymatic antioxidants such as total phenolics (PHEN) and anthocyanins (ANTH) neutralize ROS as well, thus additionally enhancing cellular tolerance to oxidative stress [40,41,42,43,44].

To integrate oxidative damage and antioxidant defense, we employed the oxidative damage/antioxidant capacity (ODAC) index, which reflects the balance between lipid peroxidation and antioxidant protection. Higher ODAC values indicate greater oxidative imbalance, whereas lower values suggest more effective redox regulation. Differences in ODAC may therefore reflect divergence in redox strategies across contrasting environmental conditions.

From an evolutionary perspective, contrasting light habitats can act as a strong selective force, promoting both phenotypic plasticity and local adaptation—two fundamental mechanisms by which plant populations cope with environmental heterogeneity [45,46]. Whereas phenotypic plasticity enables a single genotype to express different phenotypes across environments, local adaptation occurs when populations evolve genotypes that perform better in their native environment than in foreign ones [47]. These strategies are not mutually exclusive but can interact in complex ways depending on the scale of environmental variation, gene flow and the cost–benefit balance of maintaining plastic responses [48,49].

**Figure 1:**
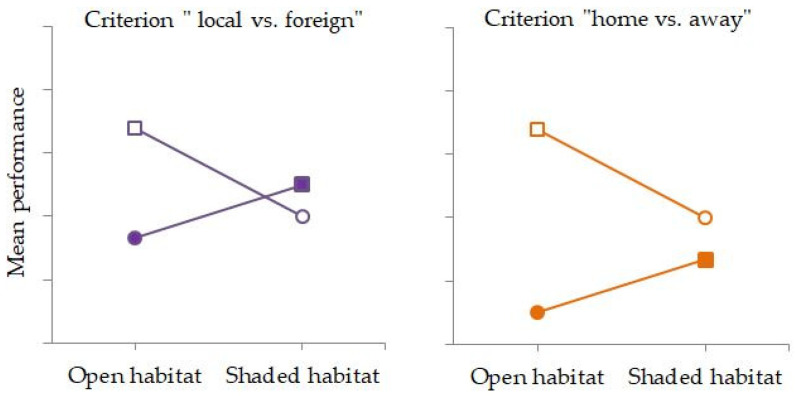
Hypothetical model illustrating the dependence of mean (trait-based) performance on the interaction between population and habitat. Mean performance of the population from the open habitat (open circles and squares) and from the shaded habitat (filled circles and squares). According to the “local vs. foreign” criterion, mean performance is compared between different populations within the same habitat—“local” (squares) and “foreign” (circles). According to the “home vs. away” criterion, fitness values of the same population are compared across habitats, that is, “at home” (squares) versus “away” (circles). Note: Although these criteria are classically defined for fitness [45], in the present study, they are used to illustrate the comparison logic applied to trait-based performance indicators, as direct fitness components (survival/reproduction) were not measured.

Reciprocal transplant experiments are generally considered a standard framework for evaluating hypotheses of local adaptation [45]. This approach involves transplanting individuals from multiple populations among their natural habitats and, within a single generation, either comparing their performance in the same habitat (“local vs. foreign” criterion) or comparing the performance of the same population across alternative habitats (“home vs. away” criterion) (Figure 1).

In the present study, the reciprocal transplant design was used to disentangle population differentiation and environmentally induced phenotypic plasticity under contrasting light conditions. Because direct fitness components were not measured, our focus was on trait expression and trait-based performance rather than on a formal test of local adaptation based on relative fitness differences. In this broader context, meta-analyses indicate that local advantage is common but not universal and that the outcomes depend on context, scale and the fitness component measured [50,51].

Understanding how plant populations respond to heterogeneous light environments is particularly important in the context of rapid anthropogenic changes in natural habitats, including land-use shifts, habitat fragmentation and alterations in canopy cover, which strongly influence light availability. In this context, *Iris pumila*, a clonal perennial naturally occurring across open and shaded habitats, represents a suitable system for examining population-level responses to contrasting light conditions. Although irradiance represents a major contrasting component between open dune and shaded woodland habitats, these environments also differ in other abiotic conditions, including temperature regimes, soil moisture dynamics, and nutrient availability, which may interact with light in shaping plant responses. By integrating leaf-level structural traits, pigment composition and antioxidant responses, this study aims to contribute to a more comprehensive understanding of plant acclimation strategies and has practical implications for the conservation of species in dynamic and fragmented landscapes.

Using a reciprocal transplant experimental design, we investigated how two natural populations of *I. pumila*—one originating from an exposed dune site and the other from a shaded woodland habitat—respond to contrasting light conditions characteristic of their native environments. Specifically, we focused on (1) the influence of light environment on the expression of structural, physiological and biochemical traits; (2) population differences in oxidative balance and antioxidant-related traits across habitats; and (3) the extent to which trait variation across contrasting light habitats is driven by phenotypic plasticity versus population-level differentiation.

## 2. Results

### 2.1. Structural, Physiological and Biochemical Leaf Traits of I. pumila in Contrasting Light Habitats

To examine the functional responses of *I. pumila* to variation in light availability, structural, physiological and biochemical leaf traits were assessed in *I. pumila* genotypes originating from two natural populations (Dune and Woods) grown in open and shaded habitats. The mean trait values with corresponding standard errors and coefficients of variation are presented in Table 1.

The examined phenotypic traits varied both between habitats within the same population and between populations within the same habitat (Table 1). In both populations, plants grown under shaded conditions generally exhibited higher mean values of leaf traits associated with light harvesting, such as SLA and photosynthetic pigments, compared to those grown in the open habitat. In contrast, plants from open habitats showed increased LDMC as well as higher POD and GR specific activities (Table 1). In both populations, the Chl *a*/*b* ratio was higher in shaded compared to open habitats, with an increase of approximately 8% in the Dune population and 13% in the Woods population. This pattern contrasts with the commonly reported decrease in Chl *a/b* under shade and reflects a proportionally greater reduction in Chl *a* in sun-exposed leaves relative to Chl *b* (Table 1).

Within the open habitat, which represents the local environment for the Dune genotypes, mean values of SLA, antioxidant enzyme activities, and ANTH tended to be higher in foreign Woods genotypes than in the local ones (Table 1). The water-related physiological trait, SLWC, exhibited similar mean values in both populations. In the shaded habitat, mean values of most examined leaf traits were generally comparable between local and foreign genotypes. However, GR activity and ANTH were found to be higher in local Woods genotypes than in the foreign ones (Table 1).

### 2.2. Phenotypic Plasticity of Structural, Physiological and Biochemical Leaf Traits to Light Intensity

**Figure 2:**
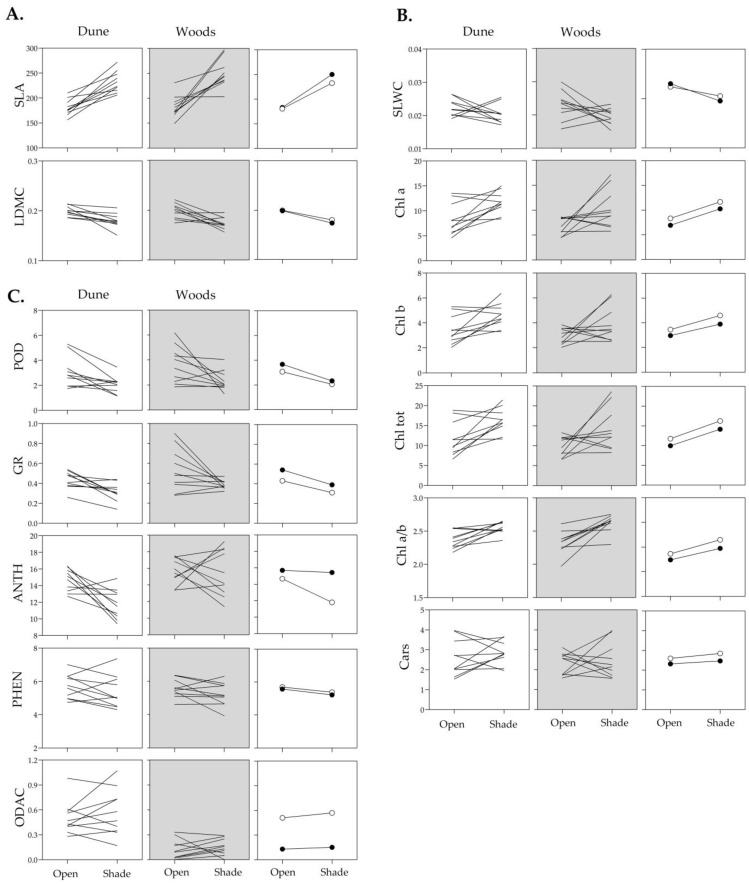
Testing the “local vs. foreign” hypothesis of local adaptation in natural populations of *I. pumila*. Reaction norms of structural (**A**), physiological (**B**) and biochemical (**C**) leaf traits in *I. pumila* genotypes from the Dune and the Woods populations, and their mean values (Dune—open symbols, Woods—closed symbols), expressed at open and shaded light habitats during summer in the Deliblato Sands. For clarity and readability, individual genotype lines are not labeled. For trait acronyms and measurement units see Table 1.

**Figure 3:**
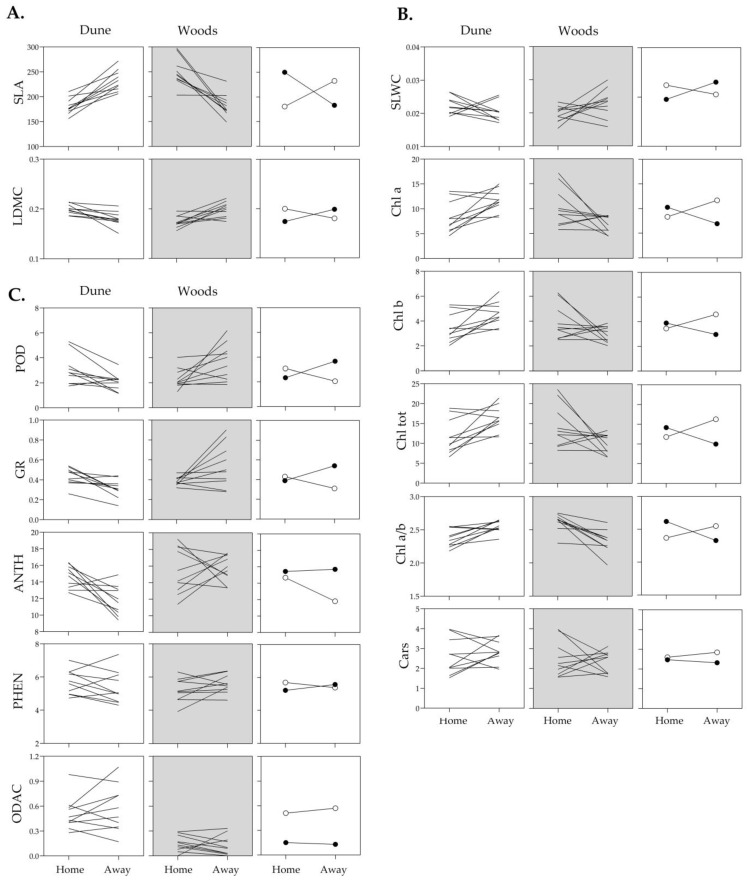
Testing the “home vs. away” hypothesis of local adaptation in natural populations of *I. pumila*. Reaction norms of structural (**A**), physiological (**B**) and biochemical (**C**) leaf traits in *I. pumila* genotypes from the Dune and the Woods populations, and their mean values (Dune—open symbols, Woods—closed symbols), expressed at open and shaded light habitats during summer in the Deliblato Sands. For clarity and readability, individual genotype lines are not labeled. For trait acronyms and measurement units see Table 1.

The reaction norm plots for all analyzed leaf traits of individual genotypes, ten from each of the Dune and Woods populations, expressed in open and shaded habitats during summer, together with the corresponding population mean reaction norms, are presented in Figure 2 and Figure 3. These plots provide a clear overview of how each trait responds to different light conditions, highlighting both general patterns and subtle differences among individual genotypes. The slopes of the reaction norms across all analyzed traits clearly demonstrated their inherent capacity of genotypes to plastically respond to changes in ambient light, with response patterns being genotype- and trait-specific in both magnitude and direction. For example, the reaction norms for SLA and photosynthetic pigments generally exhibited an upward trend with decreasing light intensity, consistently across most genotypes, while reaction norms for SLWC and biochemical traits were predominantly downward directed (Figure 2). In addition, variation in slope among individual genotypes, including frequent crossing of reaction norms and occasional rank shifts, was evident for nearly all traits within each trait-specific reaction norm, indicating the presence of genetic variation in phenotypic plasticity within both *I. pumila* populations.

This genotypic variation was further supported by coefficient of variation (CV%) analyses, which revealed habitat-, population- and trait-dependent patterns of individual variability (Table 1). Structural traits, particularly LDMC, were the most stable, showing the lowest CV% across both populations and habitats (Dune: 4.9% and 8.1% in open and shaded habitats, respectively; Woods: 7.6% and 6.7% at open and shaded habitats, respectively). Intermediate levels of variation were observed for SLWC (11.9–18.6%) and non-enzymatic antioxidants (ANTH: 9.3–18.1%; PHEN: 10–18.2%). In contrast, chlorophylls and enzymatic antioxidants exhibited the highest individual variation, with Chl *a* reaching up to 38.7% and increases in POD up to 40.2% and in GR up to 39.7%. ODAC also showed particularly high CV% values, which is expected for a composite index integrating multiple biochemical components and mainly reflects pronounced among-genotype variability in oxidative balance. As a general trend in both light environments, the percentage of individual variation tended to be smaller in local genotypes compared to foreign ones, although exceptions were observed. For instance, photosynthetic pigments consistently varied more in local genotypes under both light environments, while SLA, POD and ANTH showed greater variation in the Woods population under shade and PHEN in Dune under full sun.

Results obtained from an *F*-test of equality of variances confirmed that CV% for Chl *a* and GR differed significantly between the Dune and Woods population across both habitats (for Chl *a*: *F* = 3.48, *p* = 0.048 and *F* = 0.24, *p* = 0.029, at open and shaded habitats, respectively; for GR: *F* = 0.19, *p* = 0.015 and *F* = 6.49, *p* = 0.007, at open and shaded habitats, respectively), as well as for ODAC in the shaded habitat (*F* = 9.16, *p* = 0.002 and *F* = 6.49, *p* = 0.007). In the Dune population, the CV% values differed significantly between light environments for POD (*F* = 4.44, *p* = 0.025) and ODAC (*F* = 0.17, *p* = 0.012), whereas in the Woods population, significant differences between habitats were found for SLWC (*F* = 0.08, *p* = 0.001), GR (*F* = 0.03, *p* < 0.0001), ANTH (*F* = 3.63, *p* = 0.043), Chl *a* (*F* = 5.49, *p* = 0.013), Chl *b* (*F* = 4.86, *p* = 0.019) and Chl tot (*F* = 4.85, *p* = 0.019).

These variance patterns were reflected in the shape and range of reaction norms. Photosynthetic pigments in both populations showed greater variance in their native habitats (open for Dune and shaded for Woods), indicating that expression was strongly influenced by local light conditions (Figure 3). In contrast, the reaction norms of enzymatic antioxidants in Woods genotypes showed more variance under high light, whereas non-enzymatic antioxidants in Dune genotypes varied more under shade. Despite overall parallelism in mean reaction norms, traits such as SLA, SLWC, Chl *a*/*b* ratio and ANTH exhibited clear population-specific differences in slope and range, pointing to divergent modulation of plasticity (Figure 2).

Quantitative values of phenotypic plasticity for all traits across contrasting light environments are shown in Table 2. Plasticity varied significantly among traits, while the population effect was negligible. The highest plasticity was recorded for enzymatic antioxidants (POD and GR) and photosynthetic pigments, whereas non-enzymatic antioxidants exhibited much lower plastic potential. Particularly low plasticity values were recorded for LDMC and the Chl *a*/*b* ratio. The Wilcoxon test revealed similar plasticity levels between populations for all traits, except ODAC, which differed significantly (*p* = 0.002) (Table 2). Plasticity indices were generally higher in the Woods population, except for GR, ANTH and PHEN, where Dune genotypes showed greater plasticity. Notably, the ODAC plasticity index was about 70% greater in Dune genotypes.

To assess potential costs of plasticity, associations between trait plasticity indices and mean SLA (mSLA), used as a performance proxy, were analyzed. Multivariate regression analysis revealed no significant relationship between plasticity and mSLA, indicating no detectable cost of plasticity for any trait (Table S1). However, several individual traits showed significant effects on mSLA in both populations. In the Dune population, mSLA showed negative associations with GR, POD, ANTH, SLWC and LDMC, and a positive association with the Chl *a*/*b* ratio. In the Woods population, mSLA showed negative associations with POD, SLWC and LDMC, and a positive association with the Chl *a*/*b* ratio. These results indicate that, although plasticity *per se* did not impose a measurable cost, particular structural, physiological and biochemical traits may influence growth-related performance under contrasting light conditions. Since the regression was performed on standardized data, the magnitude of standardized coefficients (estimates) allows direct comparison among traits. The absolute values of these coefficients were generally higher in the Woods population than in the Dune population, suggesting that trait-performance associations may be stronger under shaded forest conditions.

As a measure of plasticity integration, Spearman’s correlation coefficients (ρ) among plasticity indices were calculated (Table S2). Six significant correlations were found in the Dune population (two positive, four negative), while three were detected in the Woods population (one positive, two negative). This pattern suggests potential differences in trait interrelationships between populations, with Dune genotypes showing a greater proportion of inverse associations. Despite these differences, the Mantel test revealed a significant positive correlation between the two correlation matrices (r = 0.60, *p* = 0.001), suggesting overall structure similarity in correlation structure.

**Figure 4:**
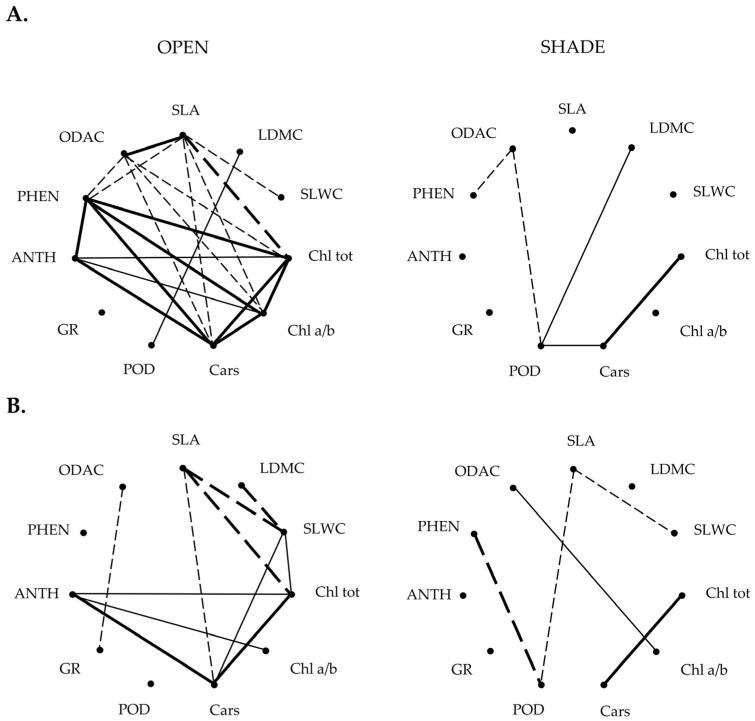
Pearson’s coefficients (r) of genotypic correlations between the structural, physiological and biochemical leaf traits in *I. pumila* genotypes from the Dune (**A**) and the Woods (**B**) populations expressed at open and shaded light habitats in the Deliblato Sands. Only statistically significant correlations are shown (*p* < 0.05). Significant positive correlations are illustrated with solid lines, whereas significant negative correlations are illustrated with dashed lines. The strength of correlations is presented by line thickness: thick line, r > 0.80; thin line, 0.60 < r < 0.80. For trait acronyms and measurement units see Table 1.

In addition to plasticity indices, relationships between mean trait values were analyzed to explore patterns of trait covariation within populations (Table S3, Figure 4). Given the relatively small number of genotypes per group (*n* = 10), these correlation analyses are interpreted as exploratory assessments of trait integration patterns. The resulting correlograms suggested differences in correlation structure across populations and habitats. In the open habitat, the Dune genotypes exhibited 21 significant associations (12 positive, 9 negative), while the Woods genotypes showed 11 (6 positive, 5 negative). Under shaded conditions, Dune genotypes displayed a much simpler correlation structure, with only 5 significant correlations, whereas Woods genotypes exhibited 11 (Figure 4).

Beyond these general patterns, some correlations were consistently observed across all populations and habitats, while others were specific to particular populations or environmental conditions. Among the consistently detected correlations, a positive one between Chl tot and Cars was observed, reflecting coordinated variation in major photosynthetic pigments. In contrast, several correlations were restricted to particular populations or habitats. For example, correlations between ODAC and PHEN, as well as between LDMC and POD, were found only in the Dune population. In the open habitat, SLA showed negative correlations with SLWC, Chl tot and Cars, whereas ANTH was negatively associated with both the Chl *a*/*b* ratio and Cars. These patterns may reflect coordinated adjustments in leaf structure and pigment composition under high-light conditions.

To evaluate whether these population- and habitat-specific patterns reflect broader structural differences in trait interrelationships, Mantel tests were performed to compare correlation matrices across populations and light environments. Significant positive correlations were detected between all matrix pairs: Dune vs. Woods in the open habitat (r = 0.583, *p* = 0.001), Dune vs. Woods in shade (r = 0.397, *p* = 0.007), Dune open vs. Dune shade (r = 0.404, *p* = 0.018) and Woods open vs. Woods shade (r = 0.577, *p* = 0.001). Altogether, these findings suggest that, despite local variation in the number and strength of trait associations, the overall structure of trait interrelationships may be conserved across populations and light environments.

### 2.3. Population Differentiation and Trait Responses Across Contrasting Light Habitats

To examine the effect of light conditions and population origin on structural, physiological and biochemical leaf traits, a two-way ANOVA was applied. Structural traits (SLA and LDMC) differed significantly between habitats (*p* < 0.0001), while population and interaction effects were not significant (Table 3).

In both populations, plants grown in shaded conditions exhibited higher SLA and lower LDMC compared to those in open habitats, reflecting typical shade-adaptive structural leaf changes. Among physiological traits, significant habitat effects were detected for all traits, except Cars, which followed a similar trend as chlorophylls but without statistical significance. No significant population or habitat × population interaction effects were observed, indicating that both populations responded similarly in their physiological traits to differing light availability. For biochemical traits, POD, GR and ANTH differed significantly between habitats, while GR, ANTH and ODAC varied significantly among populations. Specifically, GR and ANTH showed population-level differences under shaded habitats, whereas ODAC differed between populations under exposed conditions. A habitat × population interaction was detected for ANTH (*p* = 0.04), suggesting potential population-specific differences in anthocyanin plasticity (Table 3). Overall, habitat emerged as the primary source of variation for structural and physiological traits, while both habitat and population influenced variation in biochemical traits.

To test the “home vs. away” hypothesis, i.e., to examine the effect of habitat light conditions on leaf traits within each population, a one-way ANOVA was performed. These comparisons are trait-based and are interpreted as differences in functional performance indicators rather than direct fitness advantages. In the Dune population, habitat significantly affected both structural traits (SLA and LDMC), as well as photosynthetic pigments (Chl *a*, Chl *b*, Chl tot and the Chl *a*/*b* ratio), whereas Cars did not differ significantly between habitats. This pointed to a consistent structural and physiological response to light availability. Significant differences were also observed for some biochemical traits, such as POD, GR and ANTH, while PHEN and ODAC remained unchanged (Table 4; Figure 3).

In the open habitat, i.e., “home” conditions, *I. pumila* genotypes from the Dune population exhibited increased POD and GR activities, along with increased ANTH and LDMC, suggesting a greater investment in protective and structural traits under high irradiance. In the Woods population, habitat had significant effects on structural traits (SLA, LDMC), most photosynthetic pigments (Chl *a*, Chl tot, and Chl *a*/*b* ratio) and biochemical traits (POD and GR) (Table 4; Figure 3). In the shaded habitat, SLA, Chl *a*, Chl tot, and Chl *a*/*b* ratio were higher “at home” than “away”, i.e., in the open habitat. Conversely, LDMC, SLWC and specific activities of POD and GR were greater in the open habitat (Table 4; Figure 3). Taken together, these results suggest trait patterns consistent with home-site effects for particular suites of leaf traits and with population-level differentiation under contrasting light environments.

To examine population-level differentiation within the same habitat (“local vs. foreign” comparison), one-way ANOVAs were used to compare Dune and Woods genotypes within each light environment (Table 5). In the open habitat, significant differences were found only for ODAC, with Dune genotypes exhibiting higher values (Table 5; Figure 2). In the shaded habitat, population-level differences were observed not only for ODAC but also for GR and ANTH, suggesting greater differentiation in antioxidant-related traits under low-light conditions. Unlike the “home vs. away” comparison, which revealed broad habitat-driven shifts across leaf traits, the “local vs. foreign” comparisons indicated more subtle population differentiation, primarily in biochemical traits. ODAC values were consistently higher in Dune genotypes, whereas GR and ANTH levels were higher in the Woods genotypes (Table 5; Figure 2), reflecting divergent antioxidant strategies between populations under identical light conditions. This population-level pattern was obtained when ODAC was calculated using global normalization across all samples, confirming that the observed divergence is robust to the scaling approach.

## 3. Discussion

### 3.1. Leaf Functional Responses to Light Environments

Light acts not only as a primary energy source for photosynthesis, but also as a major environmental factor shaping leaf structure, physiology and biochemistry [52,53,54,55]. In this study, *I. pumila* genotypes originating from contrasting light environments exhibited distinct structural, physiological and biochemical responses when grown in habitats differing in light availability.

The observed variation in leaf structural traits and pigment-related physiological traits across contrasting light environments suggests a coordinated acclimation strategy aimed at optimizing resource use and photoprotection [56,57]. The consistent increase in SLA under shaded conditions indicates an investment in thinner, less dense leaves that maximize light capture efficiency under limited irradiance [11,21]. Conversely, higher LDMC values recorded in sun-exposed individuals reflect the formation of more compact and structurally reinforced tissues resistant to desiccation and mechanical stress, typical of plants adjusted to high evaporative demand and potential photodamage [58,59,60]. The marked increase in chlorophylls, including Chl *a*, Chl *b* and Chl tot under shade, supports the idea that shade-acclimated leaves compensate for reduced photon flux density by expanding their photosynthetic pigment pool to maximize light-harvesting capacity [61,62,63]. Contrary to classical expectations of a reduced Chl *a*/*b* ratio in shade-acclimated leaves, our results showed a significant increase in the Chl *a*/*b* ratio in both populations under shaded conditions. This shift appears to result from a proportionally stronger reduction in Chl *a* in sun-exposed leaves relative to Chl *b* (32% versus 24% lower in unshaded compared to shaded leaves), rather than from a classical shade-driven increase in Chl *b*. Similar patterns have been reported previously for *I. pumila* [62] and, although less frequently documented, have also been observed in other shade-tolerant species [64,65], suggesting that adjustments in chlorophyll composition under shade may follow multiple acclimation pathways. However, as photosystem organization, light-harvesting complex (LHC) abundance and chlorophyll fluorescence parameters were not assessed, the mechanistic interpretation of this shift remains tentative. Simultaneously, SLWC tended to be lower in shaded individuals, likely reflecting thinner, less water-dense leaves with reduced structural investment [66]. Such a pattern suggests coupling between water balance, structural investment and pigment composition in *I. pumila* leaves, enabling efficient light use without excessive water retention. By contrast, carotenoid concentrations did not differ significantly between light habitats, suggesting limited responsiveness under our experimental conditions. Given their dual role in photoprotection and structural stabilization of pigment–protein complexes, this pattern may reflect buffering of carotenoid pools, but it should not be interpreted as evidence of inherent stability [67,68,69,70,71].

Beyond these structural and physiological adjustments, *I. pumila* also exhibited clear biochemical adjustments to contrasting light environments. Elevated activities of antioxidant enzymes such as POD and GR in sun-exposed individuals indicate reinforcement of oxidative defense pathways to counteract ROS formation under high irradiance [72,73,74]. GR, for instance, has been shown to maintain *Arabidopsis* photosystem II function under excess light by preventing hydrogen peroxide accumulation [75]. Non-enzymatic antioxidants, including anthocyanins and phenolics, also contributed to light-dependent responses. Higher anthocyanin concentrations in sun-exposed plants suggest that these pigments serve as inducible photoprotective compounds, mitigating photooxidative stress and supporting redox homeostasis [72,76,77,78]. The slightly elevated anthocyanin levels in the Woods population, even within open habitat, may reflect subtle metabolic adjustments associated with long-term exposure to the more heterogeneous forest microenvironment. Such microhabitat-driven divergence is consistent with expectations of the habitat heterogeneity hypothesis [79]. Total phenolic content in *I. pumila* showed little variation between habitats or populations, implying a constitutive antioxidant function rather than an inducible one. Their stability across light environments highlights their role in maintaining baseline oxidative protection [43,64,65]. Hence, phenolics likely provide a permanent defense layer, while anthocyanins and antioxidant enzymes operate as dynamic, light-inducible mechanisms, together forming a robust multi-layered photoprotective system.

To better capture the balance between oxidative damage and antioxidative defense, we introduced the Oxidative Damage to Antioxidant Capacity (ODAC) index—a composite metric integrating lipid peroxidation (MDA) with both enzymatic (POD, GR) and non-enzymatic (PHEN, ANTH) antioxidant components—which is expected to exhibit substantial among-genotype variability as an integrative index. ODAC therefore provides a more sensitive and ecologically relevant measure of oxidative balance than traditional single indicators. The consistently higher ODAC values observed in the Dune population indicate a constitutively elevated oxidative load relative to antioxidant capacity. Importantly, higher ODAC values do not imply more efficient antioxidant protection; rather, they reflect a shift in redox balance toward a higher steady-state level of oxidative turnover. Such a pattern may be consistent with a tolerance-based redox strategy, potentially shaped by long-term exposure to high irradiance and associated oxidative pressure in open habitats [72,80]. In this context, divergence in ODAC values is interpreted as population-level differentiation in redox regulation, rather than as direct evidence of superior defense efficiency or fitness advantage. Reduced POD and GR activity and lower anthocyanin levels, together with a tendency toward higher phenolic content, may indicate metabolic adjustment toward a less energetically costly, phenolic-based antioxidant strategy under sustained light stress.

It should be acknowledged that the open dune and forest understory habitats differ in multiple environmental factors beyond light availability. Accordingly, the observed trait differences are interpreted as habitat-associated patterns rather than effects exclusively attributable to light intensity.

### 3.2. Phenotypic Plasticity to Light Intensity

Phenotypic plasticity is a fundamental mechanism that enables plants to cope with heterogeneous and fluctuating environments, particularly in response to light variability [3,81,82,83]. In this study, *I. pumila* genotypes exhibited pronounced plasticity in structural, physiological and biochemical leaf traits, emphasizing their capacity to modulate functional phenotypes in response to variation in light availability.

Quantitative patterns of phenotypic plasticity revealed clear functional differentiation among leaf traits, while differences between populations were generally limited. Enzymatic antioxidants and photosynthetic pigments exhibited the highest plasticity, suggesting that biochemical and physiological processes directly involved in stress mitigation and photosynthetic performance are particularly responsive to variation in light availability [83,84]. This pronounced responsiveness highlights the adaptive significance of maintaining flexible physiological control under fluctuating irradiance. In contrast, structural traits displayed lower plastic potential, likely reflecting constructional constraints and greater inherent stability [49,85,86]. Although overall plasticity did not differ significantly between populations, ODAC showed marked population-specific variation, with significantly greater plasticity observed in the Dune genotypes. This pattern may indicate a more dynamic redox regulation in Dune genotypes, potentially reflecting a greater redox flexibility associated with photoprotective adjustment under high irradiance [87,88,89]. Conversely, the consistently low ODAC values in Woods genotypes suggest maintenance of lower oxidative load, typical of shade-adapted species [90].

Correlations among trait plasticities provide insight into their functional integration and co-regulation under variable environmental conditions, likely reflecting shared environmental sensitivity, functional roles or genetic control [3,49,81,91]. The presence of both positive and negative correlations observed in this study suggests a balance between coordinated trait responses and functional trade-offs, although these patterns should be interpreted cautiously given the limited sample size. For example, the negative relationship between SLA and total chlorophyll observed in both populations may indicate a constraint in the plasticity of leaf structure and physiology. Likewise, the negative associations of anthocyanins with chlorophylls and carotenoids in the Dune population may reflect light-associated trade-offs under high irradiance. Conversely, the positive correlation between carotenoid and total chlorophyll plasticity in the Woods population could suggest coordinated pigment adjustments under lower light conditions [92].

In addition to differences in mean plasticity, the extent of individual variation in trait expression (measured as the coefficient of variation) provided further perspective on population responses. Structural traits showed the lowest variability, while enzymatic antioxidants and chlorophylls exhibited higher CV% values, particularly in foreign genotypes. This pattern may be consistent with greater trait stability of local genotypes under familiar conditions, whereas foreign genotypes exhibited increased variability, potentially reflecting context-dependent plastic responses in novel environments [93].

Importantly, no detectable costs of plasticity, with respect to the performance proxy used in the present study, were found, suggesting that *I. pumila* can maintain flexible phenotypic expression without compromising growth-related performance across contrasting light environments. However, this result should be interpreted cautiously. The existing literature recognizes that plasticity can involve both costs and limits and that such costs may be subtle and difficult to detect empirically [47]. Contemporary frameworks further emphasize strong context dependence and note that reported “costs of plasticity” may be difficult to separate from costs of phenotype rather than plasticity *per se* [49]. More generally, plastic responses should be interpreted in light of the ecological context and how reliably environmental cues reflect underlying heterogeneity [94].

### 3.3. Population Differentiation Under Spatial Heterogeneity in Habitat Light Conditions

Reciprocal transplant experiments have long been recognized as a powerful approach for assessing local adaptation and population differentiation, as they directly compare the performance of native and non-native genotypes across contrasting environments [45]. Such an experimental design enables the evaluation of population differentiation and the potential role of spatially heterogeneous selection in shaping trait variation. Within this framework, the present study investigated how differences in light availability between open and shaded habitats affect the expression of leaf structural, physiological and biochemical traits and how these responses contribute to population-level divergence in two natural populations of *I. pumila*.

Our findings showed that habitat conditions have exerted a strong and consistent influence on structural and physiological traits, while the effect of population origin was comparatively limited. This pattern suggests that these traits are primarily shaped by environmental light availability, reflecting plastic responses to differing irradiance regimes. In contrast, biochemical traits showed a more complex pattern of variation, with significant effects of both habitat and population. Such differentiation may reflect a combination of inherent genetic divergence and fine-scale metabolic adjustments to local environmental pressures [95]. The absence of significant habitat × population interactions further indicates that both populations respond similarly to light differences, with no evidence of divergent reaction norms. Together, these results emphasize the dominant influence of habitat conditions in driving trait differentiation, while also pointing to a secondary but meaningful contribution of population-level genetic variation, particularly in biochemical characteristics.

Further analysis of trait variation—within each population across habitats and between populations within each habitat—revealed notable differences in mean trait values. In the Dune population, the most pronounced habitat-related differences were observed in structural traits, photosynthetic pigments (excluding carotenoids) and several biochemical parameters (POD, GR and anthocyanins). Higher POD and GR activities, together with increased anthocyanin and LDMC values in the open “home” habitat, indicate greater antioxidant capacity and more structurally robust leaves, features consistent with enhanced tolerance to high irradiance and oxidative stress. Although phenolic content and ODAC did not differ significantly between habitats, phenolics tended to be higher in the open habitat, supporting their known role in photoprotection and antioxidative defense [42]. These findings suggest that even subtle, non-significant shifts in metabolite levels may contribute to ecologically relevant adjustments to light conditions.

In the Woods population, trait variation between habitats suggested contrasting response strategies. Elevated Chl *a* and Chl tot levels under shaded “home” conditions are consistent with enhanced light capture in a low-irradiance environment, a hallmark of shade-adapted species [27,96]. Conversely, higher SLA, LDMC and SLWC, along with increased POD and GR activities in the open habitat, suggest a shift toward structural reinforcement and activation of antioxidant defense mechanisms. These responses are consistent with a shift toward a more resource-conservative strategy under elevated irradiance. Altogether, the contrasting responses between shaded and open environments highlight the ecological plasticity of *I. pumila*, enabling this species to maintain functional stability and performance across heterogeneous light habitats. Because the analyzed traits were selected *a priori* and are functionally related, conclusions are based on consistent patterns across trait groups rather than isolated borderline *p*-values. It should be noted that the present study includes only one population per habitat type. Therefore, our conclusions are restricted to population-level differentiation between these two populations and should not be interpreted as evidence of generalized habitat-type local adaptation across the species’ entire range. Replication across multiple populations per habitat would be necessary to evaluate broader evolutionary patterns. In addition, trait measurements were based on a single summer sampling and thus represent a single time-point assessment rather than seasonal dynamics. Moreover, fine-scale microenvironmental heterogeneity within each habitat likely contributes to within-habitat variance and should be considered when interpreting trait-level differences.

## 4. Materials and Methods

### 4.1. The Study Species and Experimental Design

*I. pumila* L. (Iridaceae), commonly known as the dwarf bearded iris, is a clonal, perennial, rhizomatous monocot native to the Eurasian Steppe belt. Its distribution ranges from Austria in the west, across Central and Southeastern Europe, to Western Siberia [97,98,99,100]. Within its natural range, the species is well represented in the Deliblato Sands (44°57’59” N 21°01’53” E)—a large continental dune complex in the southeastern Pannonian Plain, Banat, Serbia [101]. A notable ecological feature of this area is the spatial patchiness of steppe and forest habitats, both of which are inhabited by *I. pumila* populations. Dune habitats are dominated by annual and perennial herbaceous species along with low shrubs, while forest habitats consist of sandy areas afforested with black pine (*Pinus nigra*), Scots pine (*Pinus sylvestris*) and black locust (*Robinia pseudoacacia*). These habitats differ in numerous abiotic factors, with light intensity and quality being particularly pronounced [102]. The mean photosynthetic photon flux density (PPFD) and that of red: far-red (R:FR) light ratio were 1378 µmol m^−2^ s^−1^ and 1.066, at the dune open site, and 45 µmol m^−2^ s^−1^ and 0.463 under the forest shade. Measurements were made with a point quantum sensor (LI-190SA, Li-Cor, Inc., Lincoln, NE, USA). PAR and R:FR were measured approximately ten times per habitat during the summer period on the experimental plots, and the reported values represent mean estimates [72].

*I. pumila* propagates clonally through underground rhizomes that branch laterally from a primary parental rhizome. Successive branching orders (primary, secondary, tertiary and higher) form compact segments of approximately 2.5 cm in length, resulting in the formation of circular clones ranging from 30 to 120 cm in diameter, with size reflecting clone age [103]. Due to their genetic uniformity, clonal plants represent particularly suitable model organisms for ecological and evolutionary studies. Clonal propagation allows for the repeated examination of the same naturally occurring genotypes across multiple years and experimental conditions. This facilitates the collection of sufficient replicates without compromising population structure or genetic diversity [104]. Consequently, multiple ramets of the same *I. pumila* genotype can be sampled and transplanted across different environments, enabling reliable assessment of both phenotypic plasticity and local adaptation.

**Figure 5:**
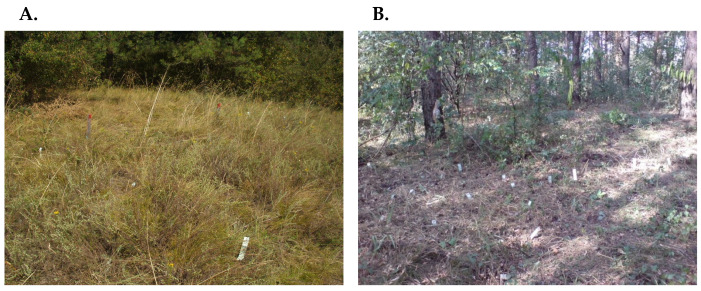
Reciprocal transplant experiment *in situ*, conducted in two contrasting light habitats: open (**A**) and shaded (**B**).

To examine population differentiation and environmentally dependent phenotypic plasticity in natural populations of *I. pumila*, a reciprocal transplant experiment was established in the Deliblato Sands (Figure 5). This experimental design enables the evaluation of population–habitat interactions according to two criteria: (1) the “local vs. foreign” criterion, which compares multiple populations within the same habitat, with the expectation that the local population will exhibit higher fitness; and (2) the “home vs. away” criterion, which compares the performance of a single population across habitats, expecting higher fitness in its native environment [45].

For this study, two populations naturally occurring in contrasting light environments were selected: one from an open dune site (“Dune”) and the other from a shaded forest under the canopy of *Pinus sylvestris* (“Woods”). Prior to transplantation, rhizome modules from each population were maintained under controlled growth-room conditions for three years to minimize maternal environmental effects and standardize the physiological state of the experimental material. Growth-room conditions were maintained at 23/19 °C (day/night) under a 16 h photoperiod, with a photosynthetic photon flux density (PPFD) of 110.5 µmol m^−2^ s^−1^. Plants were grown in 500 cm^3^ pots containing a 2:1 (*v*/*v*) mixture of soil substrate and sand and were watered regularly once a week with a nutrient solution. To minimize positional effects, pots were rotated twice weekly. Subsequently, ten adult genotypes per population were used to establish paired ramets across habitats. For each genotype, one ramet was replanted in its native (“home”) habitat, and one was reciprocally transplanted into the alternative (“away”) habitat. These were maintained *in situ* for seven years prior to sampling to ensure prolonged exposure to habitat-specific environmental conditions. All transplanted genotypes survived throughout the experimental period and were included in the final analyses. A single sampling was conducted in summer, consistent with the rationale of reciprocal transplant experiments that assess population-level responses within a single generation. The summer season was chosen because it corresponds to the period of maximal physiological expression of light-sensitive traits. Two fully developed leaves were collected from each genotype, both originating from the same ramet. One leaf was used for structural and physiological analyses (SLA, LDMC and SLWC), while the second leaf was used for biochemical assays, including photosynthetic pigments, enzymatic and non-enzymatic antioxidants. Leaf selection was standardized to minimize within-plant variability. Statistical analyses were performed at the genotype level (*n* = 20), which was treated as the experimental unit. Leaves designated for structural and physiological analyses were sealed in plastic bottles with Parafilm to prevent desiccation and transported to the laboratory under cooled conditions. Leaves intended for biochemical analyses were immediately frozen in liquid nitrogen, transferred to the laboratory and stored at −70 °C until further processing.

### 4.2. Estimation of SLA, LDMC, SLWC and Photosynthetic Pigments

To estimate specific leaf area (SLA, cm^2^ g^−1^), leaf dry matter content (LDMC, g g^−1^) and specific leaf water content (SLWC, g g^−1^), leaf area, as well as leaf fresh and dry mass, were measured. The leaf area (LA, cm^2^) was determined from digital images of scanned fresh leaves (CanoScan 5600F, Canon Inc., Tokyo, Japan) processed using the Image Tool 3.0 software. Leaf fresh mass (LFM, g) was recorded immediately after scanning and leaves were then oven-dried at 60 °C to a constant weight to determine leaf dry mass (LDM, g). Leaf traits were calculated as follows [105,106]: SLA = LA/LDM; LDMC = LDM/LFM; SLWC = (LFM − LDM)/LA.

Chlorophyll *a*, chlorophyll *b*, total chlorophyll and total carotenoids were quantified following Wellburn [107]. Briefly, leaf tissues were ground to a fine powder in liquid nitrogen and extracted in DMSO (1:30 FW:V). The homogenates were incubated for 6 h at 60 °C in a thermoblock, followed by centrifugation at 10,000× *g* for 20 min. Absorbance of the supernatant was recorded in microtiter plates at 665, 649 and 480 nm. Pigment concentrations were calculated using Wellburn’s equations and expressed as μg cm^−2^. In addition to quantifying chlorophyll *a* and chlorophyll *b*, their ratio (Chl *a*/*b*) was also calculated as an indicator of plant acclimation to contrasting light environments.

### 4.3. Determination of Enzymatic Antioxidant Activity—Peroxidase and Glutathione Reductase

Peroxidase (POD) activity was assessed by monitoring the increase in absorbance at 430 nm. The reaction mixture (1 mL total volume) contained 50 μL of crude extract, 100 mM potassium phosphate buffer (pH 6.5), 6 mM pyrogallol (ε_430_ = 2.4 mM^−1^ cm^−1^) as the hydrogen donor, and 10 mM H_2_O_2_ [108].

Glutathione reductase (GR) activity was determined by monitoring the decrease in absorbance at 340 nm, resulting from NADPH oxidation [109]. The reaction mixture (1 mL) contained 50 μL of crude extract, 50 mM potassium phosphate buffer (pH 7.5), 1 mM oxidized glutathione (GSSG), 1 mM EDTA, and 0.1 mM NADPH (ε = 6.2 mM^−1^ cm^−1^).

Soluble protein content in crude extracts was determined according to the Bradford method [110], using bovine serum albumin (BSA) as a standard. Protein concentrations were calculated from a calibration curve, and all samples were measured in triplicate. Absorbance values were within the linear range of the assay, and an internal control sample (a pooled extract of all samples) was included to ensure assay consistency. Specific POD activity was expressed as µmol min^−1^ mg^−1^ soluble protein, and specific GR activity was expressed as μmol NADPH min^−1^ mg^−1^ soluble protein.

### 4.4. Determination of the Total Non-Enzymatic Antioxidants—Phenolics and Anthocyanins

Total phenolic content (PHEN) was determined colorimetrically using the method of Singleton and Rossi [111]. Leaf tissue was ground to a fine powder in a mortar and then extracted for 1 h in 80% methanol (FW:V = 1:15). After centrifugation at 10,000× *g* for 20 min, the supernatant was collected while the precipitate was re-extracted for 1 h in 80% methanol (FW:V = 1:7). Following centrifugation, the supernatants were pooled and used for further analysis. For the assay, 50 μL of leaf extract was mixed with 475 μL of 0.25 N Folin–Ciocalteu reagent, and after 3 min, 475 μL of 1 M NaCO_3_ was added. The reaction mixture was incubated at 30 °C for 1 h afterwards the absorbance was measured at 724 nm. The standard curve was generated based on different concentrations of gallic acid (0.05, 0.1, 0.2, 0.3, 0.4 and 0.5 mg mL^−1^) dissolved in 80% methanol. The total phenolic content was expressed as mg gallic acid equivalents per gram of fresh weight.

Total anthocyanin content (ANTH) was determined according to Mancinelli et al. [112]. Leaf tissue was homogenized in liquid nitrogen, followed by extraction in acidic methanol (1% HCl) for 48 h at 5° C under continuous stirring. The absorbance of the supernatant was measured spectrophotometrically in microtiter plates at 530 and 653 nm. To correct for carbohydrate interference, anthocyanin absorbance was calculated as A_530_—0.24 A_653_ [113]. The anthocyanin content was expressed as µg cyanidin-3-glucoside equivalents per gram fresh weight. The extinction coefficient of cyanidin-3-glucoside at 530 nm is 26,900 L mol^−1^ cm^−1^, with a molecular mass of 445.

### 4.5. Assessment of Oxidative Damage to Antioxidant Capacity Index

To assess the balance between oxidative damage and antioxidant capacity, an index, herein termed ODAC (Oxidative Damage to Antioxidant Capacity), was calculated as the ratio of lipid peroxidation level (MDA) to the sum of enzymatic (POD, GR) and non-enzymatic (PHEN, ANTH) antioxidants: ODAC = MDA/(POD + GR + PHEN + ANTH). Higher ODAC values indicate greater oxidative damage relative to antioxidant investment (i.e., greater oxidative imbalance), whereas lower values indicate a lower oxidative damage relative to antioxidant investment. MDA was determined from the same leaf samples used for the antioxidant analyses in the present study, collected during the same sampling event. Analytical procedures and detailed results were reported previously [114] and the corresponding values were incorporated into the ODAC calculation. All samples represented identical genotype × habitat combinations and were stored under the same conditions prior to biochemical analyses. No samples were missing or excluded from the ODAC computation.

Prior to ODAC computation, all variables constituting the index were normalized to remove scale effects and enable their direct comparison within the composite ratio. Normalization was performed using min–max scaling within each light environment, according to the formula: x′ = (x − min)/(max − min), where x represents the original value, whereas min and max correspond to the minimum and maximum values of the respective variable within each habitat group (open or shaded). This procedure rescaled all variables to the 0–1 range, and the normalized values were then used to calculate ODAC for each genotype. To verify the robustness of the index, ODAC was additionally calculated using global normalization across all samples; this alternative scaling yielded the same population-level pattern. Because normalization was performed separately within each habitat, ODAC values are interpreted primarily for within-habitat population comparisons rather than as absolute cross-habitat magnitudes. As the ODAC index represents a comparative integration of antioxidant components rather than a mechanistic model of ROS detoxification, all variables were treated as equally contributing after normalization.

### 4.6. Statistical Analyses

All statistical analyses were performed at a genotype level (*n* = 20), including 10 from the Dune population and 10 from the Woods population. Descriptive statistics (mean, standard error and coefficient of variation) were calculated using the PROC MEANS procedure in SAS 9.3 (SAS Institute Inc., Cary, NC, USA, 2011) [115]. Reaction norm plots were generated for each genotype across the two light habitats using GraphPad Prism (version 5.01, GraphPad Software, San Diego, CA, USA).

To assess the effects of light environment and population origin on structural, physiological and biochemical leaf traits, a two-way ANOVA was conducted using the GLM procedure in SAS. Prior to analysis, the normality of residuals and homogeneity of variances were examined. Normality and homoscedasticity were evaluated using standard tests implemented in SAS. When assumptions were not met, nonparametric alternatives were used (e.g., Wilcoxon rank-sum for plasticity indices). The ANOVA model included these sources of variation: habitat (differences in analyzed traits between light environments), population (genetic differences in trait means between populations) and the habitat x population interaction (differences in plasticity between populations). A significant interaction term was interpreted as evidence of population-specific responses to light, i.e., potential local adaptation [116].

To further examine whether local light conditions have selected for distinct trait responses in the open and shaded populations of *I. pumila*, the “local vs. foreign” and “home vs. away” tests proposed by Kawecki and Ebert [45] were applied. The “local vs. foreign” test compares the performance of local and foreign genotypes (i.e., genotypes from the other populations) within the same habitat, while the “home vs. away” test evaluates whether genotypes perform better in their native environment than in the alternative one. Both approaches are based on the expectation that locally adapted genotypes exhibit higher fitness at home than away and perform better than foreign genotypes under the same environmental conditions. Differences in mean trait values were examined using one-way ANOVAs to compare: (i) the Dune and Woods populations within each light habitat and (ii) each population across the two light habitats. The models were used to estimate the significance of habitat as a source of phenotypic variation, as well as to assess genetic differentiation between populations in mean leaf trait values within the same habitat. Accordingly, habitat and population were included as primary sources of variation in the respective ANOVA models.

Phenotypic plasticity in trait response to light conditions was quantified for each *I. pumila* genotype using the plasticity index, *PI*_V_, calculated following Valladares et al. (2006) [117]: *PI*_V_ = |(X_1_ − X_2_)|/|(X_1_ + X_2_)|, where X_1_ represents the trait value of a clone in the open habitat, whereas X_2_ represents the value of the same clone in the shaded habitat. This index ranges from 0 (no plasticity) to 1 (maximum plasticity). Differences in mean plasticity between the Dune and Woods genotypes were assessed using the Wilcoxon rank-sum test. To examine integration among plastic responses, Spearman’s correlation coefficients were calculated among the plasticity indices.

To test for potential costs of phenotypic plasticity, standardized regression analysis was performed (PROC REG, SAS) within each population, using mean specific leaf area (mSLA) as a performance proxy, as it reflects genotype growth potential and light-use strategy in the absence of direct fitness measures. For each trait, a separate model was fitted, including two predictors: the mean trait value and its corresponding plasticity index. Because the analyses were conducted on standardized variables, regression coefficients allow for the direct comparison of effect sizes between trait means and plasticity indices.

Genetic correlations among leaf traits were evaluated within each population and habitat using Pearson’s correlation coefficient (r). Correlations among plasticity indices were assessed using Spearman’s rank correlation coefficient (ρ) due to deviations from normality and variance heterogeneity. To avoid redundancy and mathematically induced associations, Chl *a* and Chl *b* were excluded from correlation analyses, while only Chl tot and the Chl a/b ratio were retained. All correlation analyses were conducted using the PROC CORR procedure in SAS (with the SPEARMAN option applied for plasticity indices). For network visualization, only statistically significant correlations (*p* < 0.05) were displayed, and line thickness was used to indicate correlation strength (|r|) using pragmatic cut-offs to improve readability. Differences in correlation matrices were evaluated using the Mantel test, implemented in the vegan package in R [118]. The Mantel statistic (r) and its associated *p*-value were used to assess the strength and significance of correlation pattern divergence. Traits were selected a priori, and conclusions are based on consistent patterns across trait groups rather than isolated borderline *p*-values.

## 5. Conclusions

Using a reciprocal transplant experiment, this study provides a comprehensive view of how *Iris pumila* functionally responds to spatial heterogeneity in light availability through coordinated structural, physiological and biochemical adjustments. The observed differentiation between open and shaded habitats indicates that light intensity acts as a strong environmental filter, shaping multiple layers of leaf functional organization. Across contrasting light environments, *I. pumila* genotypes exhibited pronounced variation in SLA, LDMC, SLWC and pigment content, suggesting an integrated acclimation strategy that balances light harvesting, photoprotection and water-use efficiency. Relatively stable carotenoid and phenolic contents across habitats highlight the dual role of these metabolites in constitutive protection and structural stabilization of the photosynthetic apparatus. In contrast, inducible antioxidants such as anthocyanins and antioxidative enzymes (POD, GR) function as dynamic components of defense against light-induced oxidative stress. The implementation of the Oxidative Damage to Antioxidant Capacity (ODAC) index—calculated as the ratio between oxidative damage (MDA content) and the combined antioxidant capacity of enzymatic (POD, GR) and non-enzymatic (phenolics, anthocyanins) components—provides an integrative and ecologically meaningful measure of oxidative balance by jointly considering both damage and defense aspects of redox homeostasis. Patterns of phenotypic plasticity indicate that *I. pumila* possesses substantial capacity for functional adjustment to variation in light availability. The highest plasticity observed for enzymatic antioxidants and pigments underlines the importance of physiological and biochemical flexibility in mediating responses to contrasting irradiance regimes, whereas structural traits exhibited comparatively stable responses. Importantly, the absence of detectable plasticity costs suggests that genotypes are capable of maintaining high responsiveness without measurable reductions in growth-related performance across environmental gradients, although direct fitness consequences remain to be evaluated.

Overall, the findings indicate that environmental conditions—particularly light availability—exert a stronger influence on trait expression than population origin, highlighting the predominant role of phenotypic plasticity in shaping functional variation in *I. pumila*. At the same time, the detection of population-level differences, especially in ODAC values, suggests differentiation in redox-related traits between the two studied populations. Whether such divergence translates into differential fitness under natural conditions warrants further investigation based on direct survival and reproductive measures.

## Figures and Tables

**Table 1:** The means ($\bar{✗}$), standard errors (SE), and coefficients of variation (CV%) for the structural leaf traits: specific leaf area (SLA, in cm^2^ g^−1^) and leaf dry matter content (LDMC, in g g^−1^); physiological leaf traits: specific leaf water content (SLWC, in g cm^−2^), Chlorophyll *a*, (Chl *a*, in μg cm^−2^), Chlorophyll *b* (Chl *b*, in μg cm^−2^), Chlorophyll total (Chl tot, in μg cm^−2^), Carotenoids (Cars, in μg cm^−2^); and biochemical leaf traits: specific peroxidase activity (POD, in µmol min^−1^ mg^−1^ soluble protein) specific glutathione reductase activity (GR, in μmol NADPH min^−1^ mg^−1^ soluble protein), total anthocyanin content (ANTH, in µg cyanidin-3-glucoside equivalents per gram fresh weight), total phenolic content (PHEN, in mg gallic acid equivalents per gram of fresh weight) and the Oxidative Damage to Antioxidant Capacity index (ODAC), calculated as the ratio of normalized MDA to normalized antioxidant components (POD + GR + ANTH + PHEN) in leaves of *I. pumila* genotypes (*n* = 20 genotypes) from two natural populations, an open—the Dune and a shaded—the Woods, as expressed at their native and non-native habitats.

TRAITS	OPEN HABITAT						SHADED HABITAT					
	DUNE			WOODS			DUNE			WOODS		
	$\bar{✗}$	SE	CV%	$\bar{✗}$	SE	CV%	$\bar{✗}$	SE	CV%	$\bar{✗}$	SE	CV%
*STRUCTURAL*												
SLA	180.6	5.2	9.0	183.1	7.1	12.2	232.4	6.7	9.1	250.0	9.0	11.4
LDMC	0.1996	0.0031	4.9	0.1986	0.0048	7.63	0.1801	0.0046	8.1	0.1737	0.0037	6.7
*PHYSIOLOGICAL*												
SLWC	0.0224	0.0008	11.9	0.0230	0.0013	18.6	0.0205	0.0009	13.8	0.0195	0.0008	12.3
Chl *a*	8.32	1.02	38.7	6.93	0.54	24.8	11.67	0.68	18.5	10.26	1.22	37.9
Chl *b*	3.45	0.36	33.3	2.96	0.20	21.5	4.59	0.30	20.5	3.89	0.44	36.0
Chl tot	11.77	1.38	37.1	9.97	0.78	24.6	16.26	0.97	19.0	14.16	1.68	37.5
Chl *a*/*b* ratio	2.37	0.04	5.7	2.32	0.05	7.3	2.55	0.03	3.5	2.62	0.04	4.9
Cars	2.60	0.29	34.9	2.32	0.18	23.8	2.85	0.18	20.1	2.47	0.28	36.0
*BIOCHEMICAL*												
POD	3.08	0.39	40.2	3.66	0.46	40.1	2.06	0.21	32.3	2.34	0.25	34.1
GR	0.43	0.03	19.7	0.54	0.07	39.7	0.31	0.03	29.4	0.39	0.01	11.0
ANTH	14.67	0.43	9.3	15.70	0.50	10.0	11.80	0.56	15.1	15.44	0.88	18.1
PHEN	5.69	0.24	13.1	5.57	0.18	10.0	5.38	0.31	18.2	5.21	0.22	13.6
ODAC	0.51	0.06	39.4	0.13	0.04	93.4	0.57	0.09	49.0	0.15	0.03	64.2

**Table 2:** Phenotypic plasticity indices, *PI_V_* (mean values and coefficients of variation, CV%) estimated for leaf traits of *I. pumila*. The Wilcoxon test was employed to determine the statistical significance of differences between genotypes originating from the Dune and Wood populations grown in a reciprocal-transplantation experiment in the Deliblato Sands. The *p*-values indicating the significance of these differences are as follows: ns—non significant; ** *p* < 0.01. For trait acronyms and measurement units see Table 1.

Trait	DUNE		WOODS		*p*
	*PI_V_*	CV%	*PI_V_*	CV%	
*STRUCTURAL*					
SLA	0.125	44.2	0.153	58.9	ns
LDMC	0.052	85.4	0.073	61.2	ns
*PHYSIOLOGICAL*					
SLWC	0.097	57.0	0.126	61.8	ns
Chl *a*	0.200	77.2	0.216	89.8	ns
Chl *b*	0.166	86.0	0.186	87.7	ns
Chl tot	0.189	80.4	0.209	88.5	ns
Chl *a*/*b* ratio	0.040	67.4	0.060	70.9	ns
Cars	0.143	75.5	0.186	60.7	ns
*BIOCHEMICAL*					
POD	0.213	75.0	0.228	86.9	ns
GR	0.181	76.2	0.164	91.7	ns
ANTH	0.121	72.6	0.098	55.7	ns
PHEN	0.071	40.8	0.053	99.3	ns
ODAC	0.172	56.1	0.100	57.8	**

**Table 3:** The two-way ANOVA results for the structural, physiological and biochemical leaf traits of distinct *I. pumila* genotypes, originating from the Dune and the Woods population, which were reciprocally transplanted between their local habitats in the Deliblato Sands. For trait acronyms and measurement units see Table 1.

Trait	Source					
	Habitat (df = 1)		Population (df = 1)		H × P (df = 1)	
	*F*	*p*	*F*	*p*	*F*	*p*
*STRUCTURAL*						
SLA	69.73	**<0.0001**	1.98	0.1680	1.14	0.2937
LDMC	29.25	**<0.0001**	0.82	0.3706	0.45	0.5062
*PHYSIOLOGICAL*						
SLWC	7.32	**0.0103**	0.03	0.8740	0.58	0.4511
Chl *a*	13.53	**0.0008**	2.38	0.1320	0.00	0.9906
Chl *b*	9.33	**0.0042**	3.13	0.0851	0.09	0.7602
Chl tot	11.99	**0.0014**	2.42	0.1289	0.01	0.9053
Chl *a*/*b* ratio	31.44	**<0.0001**	0.07	0.7957	1.94	0.1718
Cars	0.68	0.4150	1.89	0.1782	0.04	0.8477
*BIOCHEMICAL*						
POD	11.50	**0.0017**	1.58	0.2173	0.20	0.6610
GR	11.82	**0.0015**	5.14	**0.0295**	0.13	0.7181
ANTH	6.38	**0.0161**	14.30	**0.0006**	4.45	**0.0419**
PHEN	1.93	0.1728	0.34	0.5610	0.01	0.9300
ODAC	0.59	0.4490	45.29	**<0.0001**	0.11	0.7379

**Table 4:** Testing the “home vs. away” hypothesis of local adaptation. Results of the analysis of variance (ANOVA) testing the effects of habitat on leaf traits of *I. pumila* genotypes originating from a sun-exposed (Dune) and a shaded (Woods) population, which were reciprocally transplanted between their local habitats in the Deliblato Sands. MS = root mean square. Significant *F* values are given in boldface. For trait acronyms and measurement units see Table 1.

Trait	DUNE				WOODS			
	df	MS	*F*	*p*	df	MS	*F*	*p*
*STRUCTURAL*								
SLA	1	13423	37.61	**<0.0001**	1	22427	34.25	**<0.0001**
LDMC	1	1.9 × 10^−3^	12.25	**0.0026**	1	3.1 × 10^−3^	17.04	**0.0006**
*PHYSIOLOGICAL*								
SLWC	1	1.9 × 10^−5^	2.45	0.1352	1	5.9 × 10^−5^	4.90	**0.0400**
Chl *a*	1	56.36	7.50	**0.0135**	1	55.64	6.15	**0.0232**
Chl *b*	1	6.46	5.84	**0.0265**	1	4.31	3.65	0.0720
Chl tot	1	100.90	7.05	**0.0161**	1	87.92	5.13	**0.0361**
Chl *a*/*b* ratio	1	0.16	12.20	**0.0026**	1	0.44	19.28	**0.0004**
Cars	1	0.29	0.51	0.4859	1	0.11	0.20	0.6568
*BIOCHEMICAL*								
POD	1	5.18	5.26	**0.0341**	1	8.75	6.26	**0.0222**
GR	1	0.07	9.51	**0.0064**	1	0.11	4.81	**0.0417**
ANTH	1	41.14	16.38	**0.0008**	1	0.33	0.06	0.8025
PHEN	1	0.49	0.65	0.4305	1	0.64	1.57	0.2260
ODAC	1	0.02	0.36	0.5537	1	3.2 × 10^−3^	0.28	0.6049

**Table 5:** Testing the “local vs. foreign” hypothesis of local adaptation. Results of the analysis of variance (ANOVA) testing the effects of habitat on leaf traits of *I. pumila* genotypes originating from a sun-exposed (Dune) and a shaded (Woods) population, grown under the same habitat conditions (open and shaded sites) in the Deliblato Sands. MS = root mean square. Significant *F* values are given in bold face. For trait acronyms and measurement units see Table 1.

Trait	OPEN HABITAT				SHADED HABITAT			
	df	MS	*F*	*p*	df	MS	*F*	*p*
*STRUCTURAL*								
SLA	1	29.47	0.08	0.7846	1	1546	2.46	0.1343
LDMC	1	4.7 × 10^−6^	0.03	0.8678	1	2.1 × 10^−4^	1.21	0.2862
*PHYSIOLOGICAL*								
SLWC	1	1.7 × 10^−6^	0.14	0.7129	1	4.2 × 10^−6^	0.60	0.4472
Chl *a*	1	9.68	1.45	0.2437	1	9.99	1.01	0.3284
Chl *b*	1	1.22	1.41	0.2498	1	2.46	1.74	0.2042
Chl tot	1	16.18	1.29	0.2714	1	22.04	1.17	0.2944
Chl *a*/*b* ratio	1	0.01	0.49	0.4943	1	0.02	2.01	0.1732
Cars	1	0.39	0.69	0.4163	1	0.69	1.23	0.2813
*BIOCHEMICAL*								
POD	1	1.71	0.93	0.3472	1	0.39	0.73	0.4041
GR	1	0.05	2.06	0.1680	1	0.03	5.60	**0.0294**
ANTH	1	5.35	2.47	0.1332	1	66.45	12.09	**0.0027**
PHEN	1	0.07	0.17	0.6879	1	0.13	0.18	0.6750
ODAC	1	0.72	26.96	**<0.0001**	1	0.88	20.11	**0.0003**

## Data Availability

The original contributions presented in this study are included in the article/Supplementary Materials. Further inquiries can be directed to the corresponding author.

## Supplementary Materials

The following supporting information can be downloaded at: https://www.mdpi.com/article/10.3390/plants15071009/s1, Table S1: Regression analysis testing for plasticity cost of *I. pumila* leaf traits to high irradiance; Table S2: Correlation coefficients for plasticity indices of *I. pumila* genotypes originating from Dune and Woods populations. Table S3: Correlation coefficients for leaf traits of *I. pumila* genotypes originating from Dune and Woods populations.
