# Supplementary material for: Leaf Structural, Physiological and Biochemical Responses to Contrasting Light Environments in Iris pumila L.: Evidence from a Reciprocal Transplant Experiment
Source: Plants (Basel). 2026 Mar 25;15(7):1009. doi: 10.3390/plants15071009 (PMC13074598; doi:10.3390/plants15071009)
Supplement: Supplementary file 1 [file plants-15-01009-s001.zip › plants-4141808-supplementary.pdf]

Table S1. Regression analysis testing for plasticity cost in *I. pumila* leaf traits to high irradiance. Standardized multivariate regression coefficients (β) of leaf traits and their plasticities (*PI*) on performance proxy (mSLA) in Dune and Woods populations. SE = standard error; t = estimate divided by its standard error; *p* = significance level. Bold type indicates regression coefficients (β) significant at the $p < 0.05$ level.

Trait / *PI*	DUNE				WOODS			
	Estimate (β)	SE	t-value	*p*	Estimate (β)	SE	t-value	*p*
LDMC	-0.097	0.031	-3.11	**0.006**	-0.103	0.040	-2.53	**0.021**
SLWC	-0.092	0.032	-2.91	**0.001**	-0.155	0.029	-5.32	**<.0001**
Chl a	0.040	0.038	1.04	0.312	0.033	0.049	0.68	0.503
Chl b	0.027	0.038	0.71	0.489	0.013	0.050	0.27	0.793
Chl tot	0.036	0.038	0.94	0.359	0.026	0.049	0.54	0.599
Chl a/b ratio	0.073	0.034	2.12	**0.049**	0.111	0.040	2.80	**0.012**
Cars	-0.024	0.038	-0.61	0.550	-0.046	0.047	-0.99	0.337
POD	-0.079	0.032	-2.41	**0.028**	-0.122	0.039	-3.11	**0.006**
GR	-0.073	0.034	-2.16	**0.045**	-0.079	0.052	-1.52	0.147
ANTH	-0.110	0.027	-4.01	**0.001**	-0.061	0.045	-1.35	0.194
PHEN	-0.030	0.037	-0.81	0.428	-0.036	0.046	-0.78	0.446
ODAC	0.040	0.037	1.09	0.290	0.038	0.058	0.65	0.525
PI LDMC	-0.008	0.031	-0.27	0.788	0.023	0.040	0.58	0.570
PI SLWC	0.016	0.032	0.49	0.627	0.036	0.029	1.24	0.230
*PI*Chl a	0.030	0.038	0.78	0.449	-0.016	0.049	-0.33	0.746
PI Chl b	0.020	0.038	0.53	0.601	-0.016	0.050	-0.32	0.753
PI Chl tot	0.027	0.038	0.70	0.495	-0.017	0.049	-0.35	0.730
PI Chl a/b ratio	0.039	0.034	1.15	0.267	0.030	0.040	0.75	0.466
PI Cars	-0.002	0.038	-0.05	0.959	-0.008	0.047	-0.17	0.870
PI POD	0.038	0.033	1.18	0.256	0.003	0.039	0.09	0.932
PI GR	-0.047	0.034	-1.38	0.184	0.046	0.052	0.89	0.385
PI ANTH	-0.011	0.027	-0.43	0.670	-0.026	0.045	-0.58	0.570
PI PHEN	0.015	0.037	0.40	0.694	0.028	0.046	0.60	0.557
PI ODAC	0.017	0.037	0.47	0.645	0.005	0.058	0.09	0.933

Table S2. Spearman's correlation coefficients (ϱ) and *p*-values for plasticity indices of *I. pumila* genotypes originating from Dune and Woods populations. The lower triangular part of the table presents correlation coefficients for genotypes from the dune population, whereas the upper triangular part presents those for genotypes from the forest population. Statistically significant correlations ($p < 0.05$) are shown in bold, while marginally significant correlations ($0.05 < p < 0.10$) are shown in italics. For trait acronyms and measurement units see Table 1.

	***PI**$_V$* **SLA**	***PI**$_V$* **LDMC**	***PI**$_V$* **SLWC**	***PI**$_V$* **Chl tot**	***PI**$_V$* **Chl a/b**	***PI**$_V$* **Cars**	***PI**$_V$* **POD**	***PI**$_V$* **GR**	***PI**$_V$* **ANTH**	***PI**$_V$* **PHEN**	***PI**$_V$* **ODAC**
***PI**$_V$* **SLA**		-0.200<br>0.580	0.479<br>0.162	**-0.770**<br>**0.009**	-0.515<br>0.128	-0.491<br>0.150	0.079<br>0.829	-0.091<br>0.803	0.030<br>0.934	0.479<br>0.162	-0.371<br>0.292
***PI**$_V$* **LDMC**	-0.321<br>0.366		*-0.612*<br>*0.060*	0.152<br>0.676	-0.418<br>0.229	-0.188<br>0.603	0.176<br>0.627	0.224<br>0.533	-0.103<br>0.777	0.236<br>0.511	0.377<br>0.283
***PI**$_V$* **SLWC**	0.430<br>0.214	-0.345<br>0.328		-0.127<br>0.726	0.418<br>0.229	-0.030<br>0.934	-0.345<br>0.328	0.042<br>0.907	-0.321<br>0.366	0.273<br>0.446	**-0.802**<br>**0.005**
***PI**$_V$* **Chl tot**	**-0.624**<br>**0.050**	0.430<br>0.214	-0.430<br>0.214		*0.588*<br>*0.074*	**0.721**<br>**0.019**	0.127<br>0.726	0.345<br>0.328	-0.394<br>0.260	-0.212<br>0.556	0.055<br>0.881
***PI**$_V$* **Chl a/b**	*-0.612*<br>*0.060*	0.248<br>0.489	-0.491<br>0.150	*0.564*<br>*0.089*		0.418<br>0.229	-0.358<br>0.310	0.212<br>0.556	-0.394<br>0.260	-0.164<br>0.652	-0.340<br>0.336
***PI**$_V$* **Cars**	-0.127<br>0.726	0.054<br>0.881	0.285<br>0.425	0.491<br>0.150	0.515<br>0.128		-0.018<br>0.960	-0.115<br>0.751	0.067<br>0.855	0.091<br>0.803	-0.097<br>0.789
***PI**$_V$* **POD**	-0.321<br>0.366	**0.697**<br>**0.025**	-0.139<br>0.701	0.212<br>0.556	0.176<br>0.627	-0.200<br>0.580		*0.564*<br>*0.089*	-0.127<br>0.726	-0.212<br>0.556	*0.559*<br>*0.093*
***PI**$_V$* **GR**	-0.248<br>0.489	-0.212<br>0.556	0.479<br>0.161	-0.006<br>0.987	-0.067<br>0.855	0.054<br>0.881	0.200<br>0.580		-0.382<br>0.276	0.018<br>0.960	0.194<br>0.590
***PI**$_V$* **ANTH**	0.224<br>0.533	-0.090<br>0.803	0.139<br>0.701	**-0.745**<br>**0.013**	**-0.697**<br>**0.025**	**-0.661**<br>**0.038**	-0.103<br>0.777	-0.176<br>0.627		0.091<br>0.803	0.194<br>0.590
***PI**$_V$* **PHEN**	0.321<br>0.366	0.248<br>0.489	-0.030<br>0.934	0.261<br>0.467	0.224<br>0.533	0.370<br>0.293	-0.164<br>0.652	-0.321<br>0.366	-0.248<br>0.489		-0.371<br>0.292
***PI**$_V$* **ODAC**	0.164<br>0.652	0.297<br>0.405	0.370<br>0.293	0.333<br>0.347	0.309<br>0.385	0.527<br>0.117	-0.152<br>0.676	-0.054<br>0.881	-0.236<br>0.511	**0.624**<br>**0.043**	

Table S3. Pearson's correlation coefficients (r) and *p*-values for leaf traits of *I. pumila* genotypes originating from Dune and Woods populations, growing in open (A) and shaded (B) natural habitats. The lower triangular part of the table presents correlation coefficients for genotypes from the dune population, whereas the upper triangular part presents those for genotypes from the forest population. Statistically significant correlations ($p < 0.05$) are shown in bold, while marginally significant correlations ($0.05 < p < 0.10$) are shown in italics. For trait acronyms and measurement units see Table 1.

**A.**	**SLA**	**LDMC**	**SLWC**	**Chl tot**	**Chl a/b**	**Cars**	**POD**	**GR**	**ANTH**	**PHEN**	**ODAC**
**SLA**		0.512<br>0.131	**-0.872**<br>**0.001**	**-0.802**<br>**0.005**	-0.427<br>0.218	**-0.752**<br>**0.012**	-0.300<br>0.400	0.249<br>0.487	-0.462<br>0.178	0.378<br>0.281	-0.255<br>0.476
**LDMC**	-0.116<br>0.750		**-0.837**<br>**0.003**	-0.283<br>0.429	0.418<br>0.229	-0.322<br>0.364	0.073<br>0.841	-0.124<br>0.732	0.126<br>0.728	0.438<br>0.206	0.277<br>0.439
**SLWC**	**-0.699**<br>**0.024**	-0.427<br>0.218		**0.659**<br>**0.038**	0.079<br>0.829	**0.642**<br>**0.046**	0.034<br>0.861	-0.023<br>0.950	0.235<br>0.513	-0.446<br>0.196	0.004<br>0.991
**Chl tot**	**-0.806**<br>**0.005**	-0.028<br>0.940	0.526<br>0.118		*0.558*<br>*0.093*	**0.948**<br>**<.0001**	0.315<br>0.374	-0.269<br>0.453	**0.741**<br>**0.014**	0.052<br>0.885	0.121<br>0.740
**Chl a/b**	**-0.760**<br>**0.011**	0.071<br>0.846	0.321<br>0.365	**0.880**<br>**0.001**		0.465<br>0.176	0.271<br>0.449	-0.174<br>0.631	**0.621**<br>**0.050**	0.305<br>0.392	0.301<br>0.398
**Cars**	**-0.775**<br>**0.008**	-0.051<br>0.888	*0.540*<br>*0.107*	**0.991**<br>**<.0001**	**0.857**<br>**0.002**		0.360<br>0.307	-0.256<br>0.475	**0.838**<br>**0.003**	0.174<br>0.630	-0.009<br>0.980
**POD**	-0.202<br>0.576	**0.788**<br>**0.007**	-0.120<br>0.741	0.187<br>0.605	0.238<br>0.507	0.197<br>0.585		0.118<br>0.746	-0.178<br>0.623	0.332<br>0.349	-0.422<br>0.224
**GR**	0.135<br>0.710	-0.154<br>0.670	0.059<br>0.872	-0.255<br>0.477	-0.279<br>0.435	-0.252<br>0.483	-0.352<br>0.319		-0.178<br>0.323	0.332<br>0.349	**-0.688**<br>**0.028**
**ANTH**	-0.415<br>0.233	-0.150<br>0.679	0.316<br>0.374	**0.795**<br>**0.006**	**0.672**<br>**0.033**	**0.860**<br>**0.001**	0.119<br>0.744	-0.262<br>0.464		0.510<br>0.132	0.019<br>0.959
**PHEN**	**-0.738**<br>**0.015**	0.037<br>0.920	0.464<br>0.177	**0.890**<br>**0.001**	**0.826**<br>**0.003**	**0.907**<br>**0.0003**	0.301<br>0.399	-0.481<br>0.159	**0.812**<br>**0.004**		*-0.571*<br>*0.085*
**ODAC**	**0.843**<br>**0.002**	-0.182<br>0.615	-0.436<br>0.208	**-0.674**<br>**0.032**	**-0.749**<br>**0.013**	**-0.652**<br>**0.041**	-0.141<br>0.698	0.122<br>0.736	-0.428<br>0.217	**-0.767**<br>**0.010**	

**B.**	**SLA**	**LDMC**	**SLWC**	**Chl tot**	**Chl a/b**	**Cars**	**POD**	**GR**	**ANTH**	**PHEN**	**ODAC**
**SLA**		-0.274<br>0.443	**-0.767**<br>**0.010**	-0.446<br>0.196	0.242<br>0.500	-0.482<br>0.159	**-0.643**<br>**0.045**	0.139<br>0.702	-0.412<br>0.237	-0.140<br>0.699	0.507<br>0.135
**LDMC**	-0.223<br>0.535		-0.354<br>0.316	-0.011<br>0.977	0.280<br>0.433	-0.026<br>0.943	0.500<br>0.141	-0.132<br>0.716	-0.155<br>0.668	0.008<br>0.983	0.404<br>0.247
**SLWC**	-0.416<br>0.231	*-0.584*<br>*0.076*		0.498<br>0.143	-0.295<br>0.408	*0.551*<br>*0.099*	0.310<br>0.384	-0.292<br>0.413	0.525<br>0.119	0.335<br>0.343	*-0.593*<br>*0.070*
**Chl tot**	-0.270<br>0.451	0.160<br>0.660	0.324<br>0.361		0.474<br>0.166	**0.995**<br>**<.0001**	0.383<br>0.274	-0.278<br>0.436	0.416<br>0.232	0.486<br>0.154	-0.070<br>0.847
**Chl a/b**	0.292<br>0.414	-0.116<br>0.751	0.051<br>0.889	-0.418<br>0.229		0.440<br>0.203	-0.080<br>0.826	-0.467<br>0.174	-0.169<br>0.634	0.520<br>0.123	**0.675**<br>**0.032**
**Cars**	-0.249<br>0.488	0.230<br>0.522	0.294<br>0.409	**0.990**<br>**<.0001**	-0.322<br>0.364		0.390<br>0.265	-0.324<br>0.360	0.453<br>0.189	0.512<br>0.128	-0.068<br>0.852
**POD**	-0.110<br>0.762	**0.742**<br>**0.014**	-0.290<br>0.416	*0.574*<br>*0.083*	-0.130<br>0.720	**0.656**<br>**0.039**		-0.163<br>0.652	*0.598*<br>*0.068*	0.168<br>0.642	-0.214<br>0.552
**GR**	0.223<br>0.535	-0.220<br>0.542	0.266<br>0.458	0.251<br>0.485	-0.058<br>0.874	0.215<br>0.550	-0.209<br>0.563		-0.264<br>0.460	**-0.868**<br>**0.001**	-0.473<br>0.168
**ANTH**	-0.240<br>0.505	0.075<br>0.837	-0.127<br>0.726	*0.586*<br>*0.075*	-0.525<br>0.119	0.539<br>0.108	-0.179<br>0.620	-0.179<br>0.620		0.504<br>0.138	-0.410<br>0.239
**PHEN**	0.326<br>0.357	0.191<br>0.598	0.121<br>0.739	0.231<br>0.521	0.371<br>0.291	0.328<br>0.355	0.212<br>0.557	0.212<br>0.557	-0.361<br>0.306		0.280<br>0.433
**ODAC**	-0.114<br>0.755	-0.438<br>0.206	0.042<br>0.907	-0.511<br>0.131	0.084<br>0.817	*-0.572*<br>*0.084*	**-0.674**<br>**0.032**	-0.465<br>0.175	-0.018<br>0.960	**-0.765**<br>**0.010**	
